# Quality control of microbiota metagenomics by k-mer analysis

**DOI:** 10.1186/s12864-015-1406-7

**Published:** 2015-03-14

**Authors:** Florian Plaza Onate, Jean-Michel Batto, Catherine Juste, Jehane Fadlallah, Cyrielle Fougeroux, Doriane Gouas, Nicolas Pons, Sean Kennedy, Florence Levenez, Joel Dore, S Dusko Ehrlich, Guy Gorochov, Martin Larsen

**Affiliations:** INRA, Institut National de la Recherche Agronomique, US1367 MetaGenoPolis, 78350 Jouy en Josas, France; UMR1319 Micalis, INRA, Jouy-en-Josas, France; Sorbonne Universités, UPMC Univ Paris 06, CR7, Centre d’Immunologie et des Maladies Infectieuses (CIMI-Paris), Hôpital Pitié-Salpêtrière, 83 bd. de l’Hôpital, 75013 Paris, France; Département d’Immunologie, AP-HP, Groupement Hospitalier Pitié-Salpêtrière, F-75013 Paris, France; Inserm UMR-S1135, Centre d’Immunologie et des Maladies Infectieuses (CIMI-Paris), F-75013 Paris, France

**Keywords:** Metagenomics, Next generation sequencing, Quality control, Sampling bias, Sample size limits

## Abstract

**Background:**

The biological and clinical consequences of the tight interactions between host and microbiota are rapidly being unraveled by next generation sequencing technologies and sophisticated bioinformatics, also referred to as microbiota metagenomics. The recent success of metagenomics has created a demand to rapidly apply the technology to large case–control cohort studies and to studies of microbiota from various habitats, including habitats relatively poor in microbes. It is therefore of foremost importance to enable a robust and rapid quality assessment of metagenomic data from samples that challenge present technological limits (sample numbers and size). Here we demonstrate that the distribution of overlapping k-mers of metagenome sequence data predicts sequence quality as defined by gene distribution and efficiency of sequence mapping to a reference gene catalogue.

**Results:**

We used serial dilutions of gut microbiota metagenomic datasets to generate well-defined high to low quality metagenomes. We also analyzed a collection of 52 microbiota-derived metagenomes. We demonstrate that k-mer distributions of metagenomic sequence data identify sequence contaminations, such as sequences derived from “empty” ligation products. Of note, k-mer distributions were also able to predict the frequency of sequences mapping to a reference gene catalogue not only for the well-defined serial dilution datasets, but also for 52 human gut microbiota derived metagenomic datasets.

**Conclusions:**

We propose that k-mer analysis of raw metagenome sequence reads should be implemented as a first quality assessment prior to more extensive bioinformatics analysis, such as sequence filtering and gene mapping. With the rising demand for metagenomic analysis of microbiota it is crucial to provide tools for rapid and efficient decision making. This will eventually lead to a faster turn-around time, improved analytical quality including sample quality metrics and a significant cost reduction. Finally, improved quality assessment will have a major impact on the robustness of biological and clinical conclusions drawn from metagenomic studies.

**Electronic supplementary material:**

The online version of this article (doi:10.1186/s12864-015-1406-7) contains supplementary material, which is available to authorized users.

## Background

Analysis of human microbiota has in recent years unraveled a universe of intricate interactions between man and microorganisms with direct implications for health and disease [1-5]. A large proportion of commensal bacterial species are presently either highly fastidious or cannot be cultured *in vitro*. This has been a major obstacle to accurately describe the microbiota composition. Metagenomic analysis based on state-of-the-art next generation sequencing (NGS) along with sophisticated bioinformatics overcomes these barriers by analyzing complex samples *ex vivo*.

Quantitative metagenomic analysis creates a gene and species profile, which allows the identification and phylogenetic classification of known as well as novel genes and species. Arumugam and co-workers discovered 3 functionally distinct gut microbiota compositions designated “enterotypes” [1]. Indeed, highly diverse consortia of commensals may functionally synergize to derive energy from nutrients in a highly coordinated and efficient manner. An imbalance of gut microbiota composition has been associated with a large range of pathologies, such as obesity [2], allergy and autoimmunity [6].

Although most studies make use of bacteria rich stool samples, a range of other body habitats with a much lower bacterial load is steadily gaining interest, such as vaginal, skin, oral and nasal body habitats [7]. A recent study demonstrates that it is technically feasible to analyze microbiota composition in samples of poor genomic DNA quantity and quality, such as dental plaques of pre-historic skeletons [8]. However, it is also increasingly clear that this type of analysis is often associated with strong biases, which are difficult to discern and complicated to correct [9]. The increasing number of samples and the use of samples from sites of low microbial density augment the importance of speed and quality control of sample processing, sequencing and data analysis. A number of studies have addressed this need by developing bioinformatics tools to monitor and correct NGS errors. Errors in this context refers to direct sequence errors at the individual base level [10,11], but also the distribution and abundance of individual sequences including sequences derived from sample or technological contaminants [12-14]. We developed a novel method, which rapidly determines and quantifies the quality of metagenomic sequence distribution at the sample level. Metagenomic analysis of complex microbiota communities is particularly sensitive to errors in sequence distribution, because abundance measures of individual bacterial genes and strains are based on sequence distribution within a given sample.

The information density of bacterial genomes is higher than complex eukaryotic organisms, because they harbor much less non-coding nucleotides [15]. Moreover, bacterial genome size is tightly linked with host symbiosis. Indeed, commensals with a long history of host symbiosis generally have small genome sizes as compared to more recent bacterial symbionts [16]. The metagenome of human gut microbiota consists of approximately 1000 different bacterial genomes and therefore has a size of approximately 1 Gbp. Of note, no single bacterial strain surpasses an abundance of 0.5% of the total gut microbiota [17], emphasizing its highly diverse nature. We therefore hypothesize that contrary to genomes of individual bacterial strains [18] a metagenome of high diversity fragmented into short sequences of length k (k-mers), would be distributed uniformly if k is sufficiently small.

K-mers are regarded as strings of length k restricted to the 4-letter alphabet (A, G, C, T). They have been used to solve various problems, such as rapid comparison of DNA sequences [19], estimation of bacterial genome size [20] and phylogeny of double-stranded DNA viruses [21]. We propose to introduce an automated k-mer distribution analysis of raw DNA sequences directly downstream of the deep-sequencing analysis. Practically, we count the occurrence of all k^4^ possible k-mers in the raw metagenomics sequence dataset (palindromic k-mers are aggregated when sequencing direction is arbitrary) and evaluate their distribution using a metric based on the information theory of Shannon [22].

Here we show that k-mer distributions of good quality metagenomic sequence data of complex gut microbiota samples are equally distributed unlike genomic sequences of individual bacterial species. We furthermore demonstrate that k-mer distribution is associated with the quality of the metagenomic data. Moreover, the Shannon Entropy of the k-mer distribution predicts the rate of sequence mapping to a predefined reference gene catalogue. Our approach analysis unprocessed raw sequences and may significantly facilitate the decision making of whether to 1) recollect, 2) reprocess a sample or 3) increase number of sequence reads before continuing with more extensive analysis. Moreover, it introduces a quality metric that may help validate conclusions made from metagenomic data.

## Methods

### Faecal sample collection and processing

Faecal samples from 30 human donors were collected in dedicated hermetically closed plastic containers kept anaerobically (oxygen poor and CO_2_ rich) with activated Anaerocult® A strips (Merck Millipore, Molsheim, France). Samples were aliquoted anaerobically and cryopreserved (−80°C) within 24 hours. Microbiota from 2.5g of stool were separated from the fecal matrix on an inverse Nycodenz® gradient under anaerobic conditions as previously described [23]. The separation yielded an average of 1.59x10^11^ (95% confidence interval = [7.8x10^10^:3.2x10^11^]) purified microbial cells per sample. Undiluted as well as four 10xfold serial dilutions of microbiota were pelleted by centrifugation (3000xg for 10 minutes) and cryo-preserved as dry-pellets for subsequent DNA extraction.

### DNA extraction

Genomic DNA was extracted using two distinct but overlapping protocols for whole stool and gradient purified commensals, respectively. Whole stool samples were treated as previously described [24]. Briefly, 200 mg of faecal sample was lysed chemically (guanidine thiocyanate and N-lauroyl sarcosine) and mechanically (glass beads) followed by elimination of cell debris by centrifugation and precipitation of genomic DNA. Finally, genomic DNA was RNase treated. DNA concentration and molecular size were estimated by Nanodrop (Thermo Scientific) and agarose gel electrophoresis. Gradient purified commensal samples were treated similar to whole stool samples with the exception that DNA precipitation was performed in smaller volumes and with extra-long incubation times.

### Metagenomic library construction

Libraries were constructed according to manufactures protocol (Life Technologies). Briefly, extracted genomic DNA was sheared by sonication, size-exclusion purified by Agencourt beads (Beckman Coulter), ligated to P1 and P2 adaptor oligonucleotides with appropriate barcodes, PCR amplified (default 6 cycles for all 52 metagenomes analysed but augmented for dilution series metagenomes as indicated in Table 1) and loaded onto the flow-chip for downstream SOLiD sequencing.

**Table 1:** **DNA quantity used for serial dilution library constructions**

	**Donor #1**			**Donor #2**		
**Sample Size (10** ^**x**^ **bacteria)**	**Purified dsDNA (ng/ml)** ^**1**^	**DNA for ligation (μg)** ^**2**^	**PCR cycles**	**Purified dsDNA (ng/ml)** ^**1**^	**DNA for ligation (μg)** ^**2**^	**PCR cycles**
10	34.9	1.00	6	30.7	1.00	6
9	4.67	0.41	7	7.17	0.35	7
8	2.68	0.07	8	2.63	0.06	8
7	0.358	<0,04	9	0.356	<0,04	9
6	0.296	<0,03	10	0.228	<0,02	10

^1^Genomic dsDNA extracted from indicated number of bacteria.

^2^Amount of sheared and size purified genomic DNA utilized for ligation with P1 and P2 adaptor oligonucleotides.

### Metagenomic sequencing and data analysis

Microbiota gene content was determined by high-throughput SOLiD sequencing of total faecal DNA [25]. An average of 34.3 million ± 36 million (mean ± s.d.) and 52.6 million ± 56.8 million 35-base-long single reads were determined for each sample from 10 dilution series samples and 52 whole stool samples, respectively (a total of 3.1 Gb of sequence). Raw sequences for all dilution series samples have been deposited in the European Bioinformatics Institute (EBI) European Nucleotide Archive (ENA) under the accession number PRJEB7925. By using Bowtie (version 1.0.0) [26] an average of 4.6 million ± 3.5 million and 13.8 million ± 15.4 million reads per individual from the two groups of samples, respectively, were mapped on the reference catalogue of 3.3 million genes [4] with a maximum of 3 mismatches. Reads mapping at multiple positions were discarded and an average of 3.6 million ± 2.7 million and 13.0 million ± 14.7 million uniquely mapped reads per individual from the two sample groups, respectively, were retained for estimating the abundance of each reference gene by using METEOR software [27]. Abundance of each gene in an individual was normalized with the method coined Reads Per Kilobase per Million (RPKM) as previously described [28]. Briefly, gene abundance was determined as the number of reads that uniquely mapped to a defined gene. Subsequently, normalized gene abundances were transformed in frequencies by dividing them by the total number of uniquely mapped reads for a given sample. The resulting microbial gene profile was used for further analyses.

### Bacterial genome sequences

28 bacterial genomes from a range of species covering common human commensals were extracted from the collection of available reference genomes from NCBI (cf. Additional file 1: Table S1).

### K-mer analysis

The abundances of all overlapping k-mer sequences present in a set of whole-genome shotgun short-read sequences were counted with in-house developed C++ software (www.mgps.eu/people/fplaza/) optimized for small k, which supports colour space reads and the CSFasta file format as input. Sequence reads with missing colour cells were discarded and remaining reads were trimmed to 35 bases. K-mer analysis of bacterial genomes was conducted with Jellyfish version 1.1 (http://www.cbcb.umd.edu/software/jellyfish/). The frequencies of different k-mers at each abundance value contained in a set of sequences are plotted as a k-mer abundance histogram. A repeated sequence in a sampled genome affects the shape of these k-mer abundance spectra depending on its length and copy number. A DNA sequence of length l will contain (l – k +1) different k-mers if it does not contain repeats of length greater than k–1.

Each k-mer has a reverse complement. E.g. the complement of 4mer ATTC is GAAT. Note that some k-mers are their own reverse complement (e.g. AGCT) if and only if k is even. Since the shot-gun short-read sequencing technology applied does not differentiate according to sequence orientation, we apply a “canonical representation”, which consider k-mers and their reverse complement equivalent (e.g. the 4-mers ATTC and GAAT are grouped together).

If the same sequence occurs n times in a genome, shotgun sequencing would sample k-mers from this sequence n times more often than those that occur in a single-copy (also referred to as average read depth). Therefore, repeated sequences in the genome result in higher abundances of associated k-mers. These collections of k-mers at higher-than-normal abundances appear as multiple peaks at different positions along the x-axis of the k-mer abundance histogram.

### Hierarchical cluster analysis

Agglomerative hierarchical cluster analysis of k-mer distributions of individual bacterial genomes performed according to Ward’s minimum variance method [29] was accomplished using JMP7 software (SAS Software, NC, USA). The optimal number of clusters was identified according to the largest distance change between successive junctions of the associated dendrogram plot. Validity and reproducibility of the classification obtained with hierarchical cluster analysis was assessed using non-hierarchical k-means cluster analysis, in which the optimal number of clusters identified through hierarchical cluster analysis was pre-specified. Reproducibility of the classifications obtained with both hierarchical and non-hierarchical clustering was assessed by determination of the kappa value.

### Ethics statement

The study was conducted in accordance with the Declaration of Helsinki. Human stool samples were obtained following acquisition of the study participants’ written informed consent and the study protocol was reviewed and approved by local ethics committee of Pitié-Salpêtrière Hospital, Paris (“Les Comités de protection des personnes”).

### Statistical analysis

Spearman’s rank correlation was calculated using the R project (http://www.R-project.org, Vienna, Austria). *P*-values < 0.05 were considered statistically significant.

## Results and discussion

### K-mer distribution of complex microbiota is homogenous irrespective of bacterial composition

**Figure 1:**
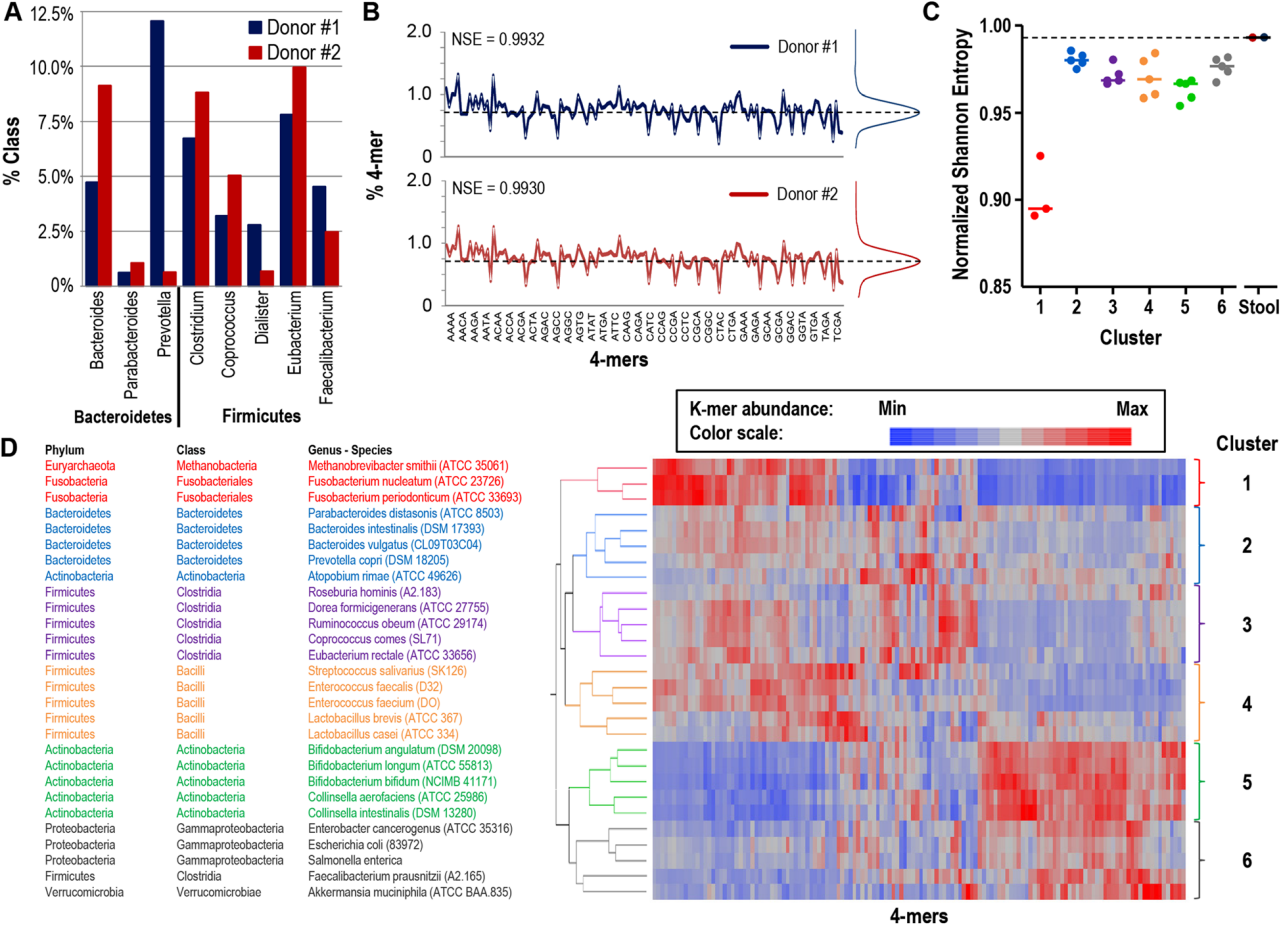
**4-mer distribution analysis for complex microbiota metagenomes compared to individual bacterial genomes. A**, Bar diagram of quantitative metagenomics of gut microbiota from two healthy volunteers, donor #1 (blue) and #2 (red), aggregated to express the frequency of a selected number of taxonomic classes from the *Bacteroidetes* and *Firmicutes* phylums. **B**, Line graph showing the 4-mer distribution of metagenomic sequences from gut microbiota of donor #1 and #2. A histogram depicting the 4-mer abundance distribution is plotted to the right of the line graph. Distribution entropy is indicated (normalized Shannon Entropy). **C**, Scatter plot visualizes the 4-mer distribution entropy for 28 bacterial genomes and two gut microbiota metagenomes. **D**, The 28 bacterial genomes are divided into 6 objective clusters by non-supervised agglomerative hierarchical cluster analysis of metagenomic 4-mer distributions based on Ward’s minimum variance method.

Highly complex microbiota metagenomic raw sequence data can be split in short sequences of length k bases, which can be binned into a finite set of possible k-mer sequences (4^k^ combinations). K-mer analysis of single bacterial genome data has previously revealed differences in k-mer distribution between bacterial species [30]. In contrast, we hypothesize that k-mer distribution of a large set of sequence data derived from a complex mix of microorganisms follows a relatively uniform distribution. To validate this hypothesis we selected two distinct stool samples representing two different enterotypes (*Prevotella* dominated for donor #1 and *Bacteroides* dominated for donor #2 - Figure 1A). We then analysed the occurrence of each 4-mer by searching through all raw sequence reads for the two metagenomes. Interestingly, the two selected metagenomes had very similar 4-mer distributions despite their highly different bacterial compositions (Figure 1B). Of note, the Shannon-Entropy for both samples was high (0.9932 and 0.9930 for donor #1 and #2, respectively) characteristic of a uniform distribution of 4-mers (Figure 1B). In line with our hypothesis, the Shannon-Entropy of the two selected metagenomes was clearly higher than the one of 28 known genomes of bacterial species from a large spectrum of phyla and classes (Additional file 1: Figure S1A top panel and C). In other words, genomes from individual bacterial species have a more heterogenous 4-mer distribution than complex metagenomes, even when such metagenomes are derived from very different gut microbiota compositions. This result was confirmed by evaluating the average normalized Shannon-index of the k-mer distribution for genomes derived from 28 bacterial strains compared to gut metagenomes derived from 21 low (<10^10^ bacteria) (cf. Additional file 1: Figure S1A middle panel) and 31 high (>10^10^ bacteria) (cf. Additional file 1: Figure S1A bottom panel) bacterial content human stool samples (*P* = 0.001 and <0.0001, respectively, cf. Additional file 1: Figure S1B). Similarly, we compared the 28 bacterial strains with 110 healthy individuals from the study by Yatsunenko *et al.* (mean and 95% confidence intervals for strains and metagenomes: 0.972 [0.963:0.980] and 0.983 [0.981:0.984], respectively, *P* = 0.004) [5]. Of note, the Yatsunenko study employed Illumina sequencing, showing that the methodology is platform-independent.

Moreover, individual bacterial genomes aggregated into 6 clusters defined by their k-mer distribution using agglomerative hierarchical cluster analysis (Figure 1D). The clusters were validated with a non-hierarchical K-means cluster analysis. The agreement between the two clustering techniques was good as defined by Cohen’s kappa agreement value (κ = 0.48). Interestingly, the identified clusters are associated with the phylogeny of the bacteria and can be used to evaluate taxonomic relations, as previously suggested [30]. Deductions from this result suggest that 4-mer analysis of metagenomes of complex bacterial mixtures can be decomposed into a linear regression of k-mer distribution vectors of individual bacteria genomes and a residual, which would represent the component unexplained by known bacterial genomes. In other words, this type of analysis could identify novel bacterial species and potentially elucidate their phylogenetic descent. This approach is beyond the scope of the present study.

### Quantitative metagenomic analysis of serially diluted gut microbiota identifies lowest analyzable sample size limit

**Figure 2:**
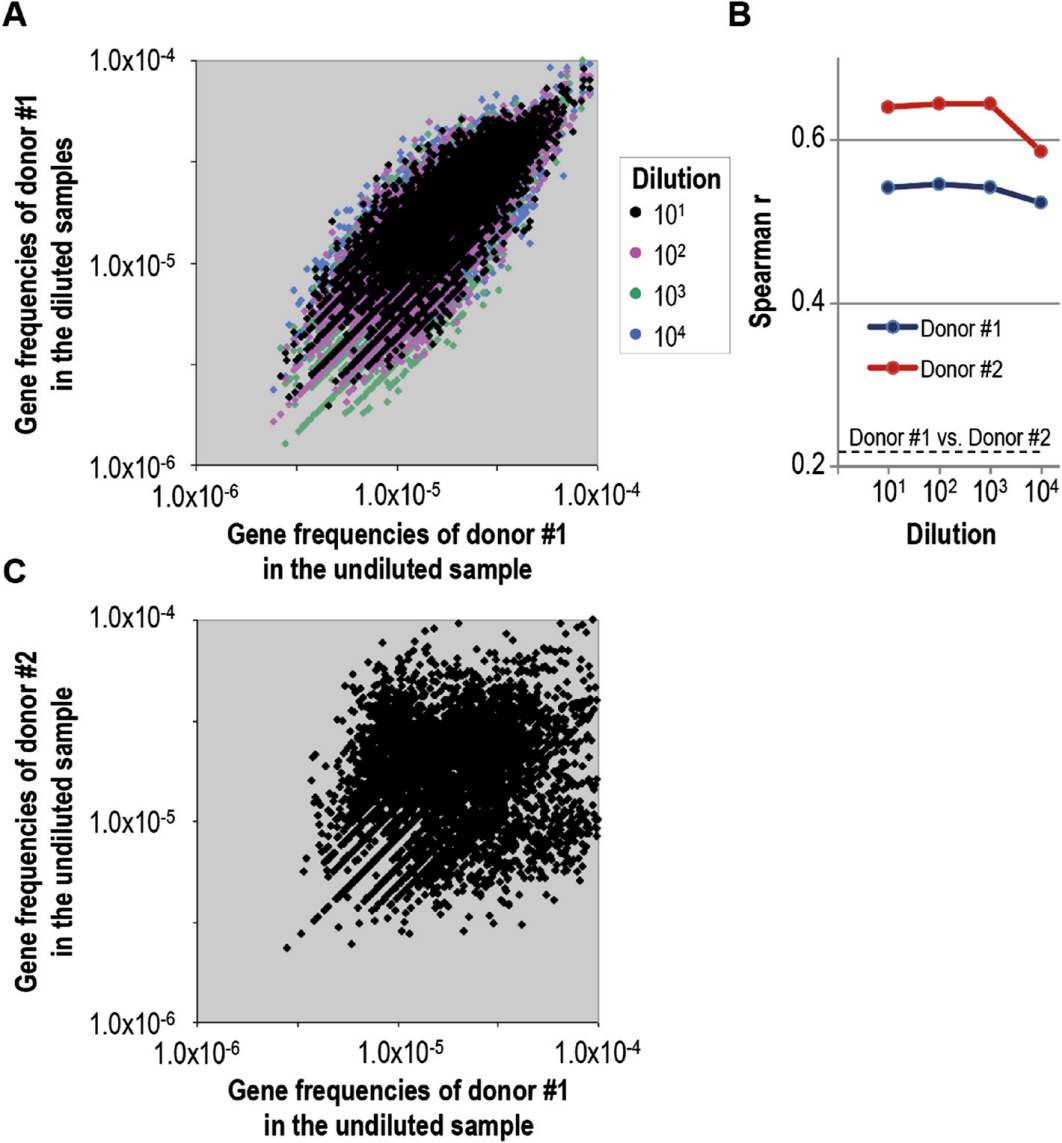
**Quantitative metagenomics of serially diluted gut microbiota. A**, Scatter plot of gene frequencies derived from quantitative metagenomic profiles of undiluted gut microbiota on the x-axis versus colour coded 10-, 100-, 1000- and 10.000-fold diluted gut microbiota on the y-axis (samples derived from donor #1 gut microbiota). **B**, Categorical line graph depicts spearman rank correlation coefficients between gene frequencies from metagenomic analysis of undiluted gut microbiota versus gene frequencies of 10-, 100-, 1000- and 10.000-fold diluted gut microbiota from donor #1 (blue) and donor #2 (red). **C**, Scatter plot of gene frequencies of undiluted samples from the two unrelated donors #1 (x-axis) and #2 (y-axis) are depicted, and their spearman rank correlation is indicated as a dotted line in B, Genes, present in the reference gene catalogue, which are not detected in the samples are excluded from the analysis.

Biased metagenomic sequence distribution can be a result of technical obstacles (DNA extraction and library construction), contaminations and limiting amount of sample material [9,31]. Whereas the former causes may be improved or avoided the latter is most often unavoidable. Of note, the reliability of sequence distribution directly affects the validity of quantitative metagenomic data. Therefore, there is an urgent need for a method to evaluate metagenomic quality. To investigate if k-mer distribution analysis of complex metagenomes could predict metagenomic quality of samples with limiting material, we generated 10-fold serial dilutions of two purified gut microbiota samples presented above (cf. Figure 1 - donor #1 and #2). Each dilution underwent genomic DNA extraction and metagenomic analysis (Table 1). All dilutions of the same sample should ideally have identical gene distribution with the more concentrated sample being the most representative of the underlying gut microbiota and thus of best quality. We therefore mapped raw metagenomic sequences onto a reference gene catalogue [4] for all analyzed samples and correlated gene frequencies from four 10-fold dilutions with gene frequencies from the most concentrated sample, serving as internal reference sample (Figure 2A). For both samples (donor #1 and #2) this analysis demonstrated strong correlations between all serial dilutions and their reference sample with a clear reduction in correlation for the highest dilution for both samples, indicating the analytical sample size limitation associated with our analytical protocol (Figure 2B). As expected, correlation between two unrelated donors (the highest concentration sample from donor #1 and #2 - spearman r = 0.22) was significantly lower than intra-donor correlations (Figure 2B and C).

### K-mer distribution analysis of metagenomic sequences identifies the same lower sample size limit as quantitative metagenomic analysis

**Figure 3:**
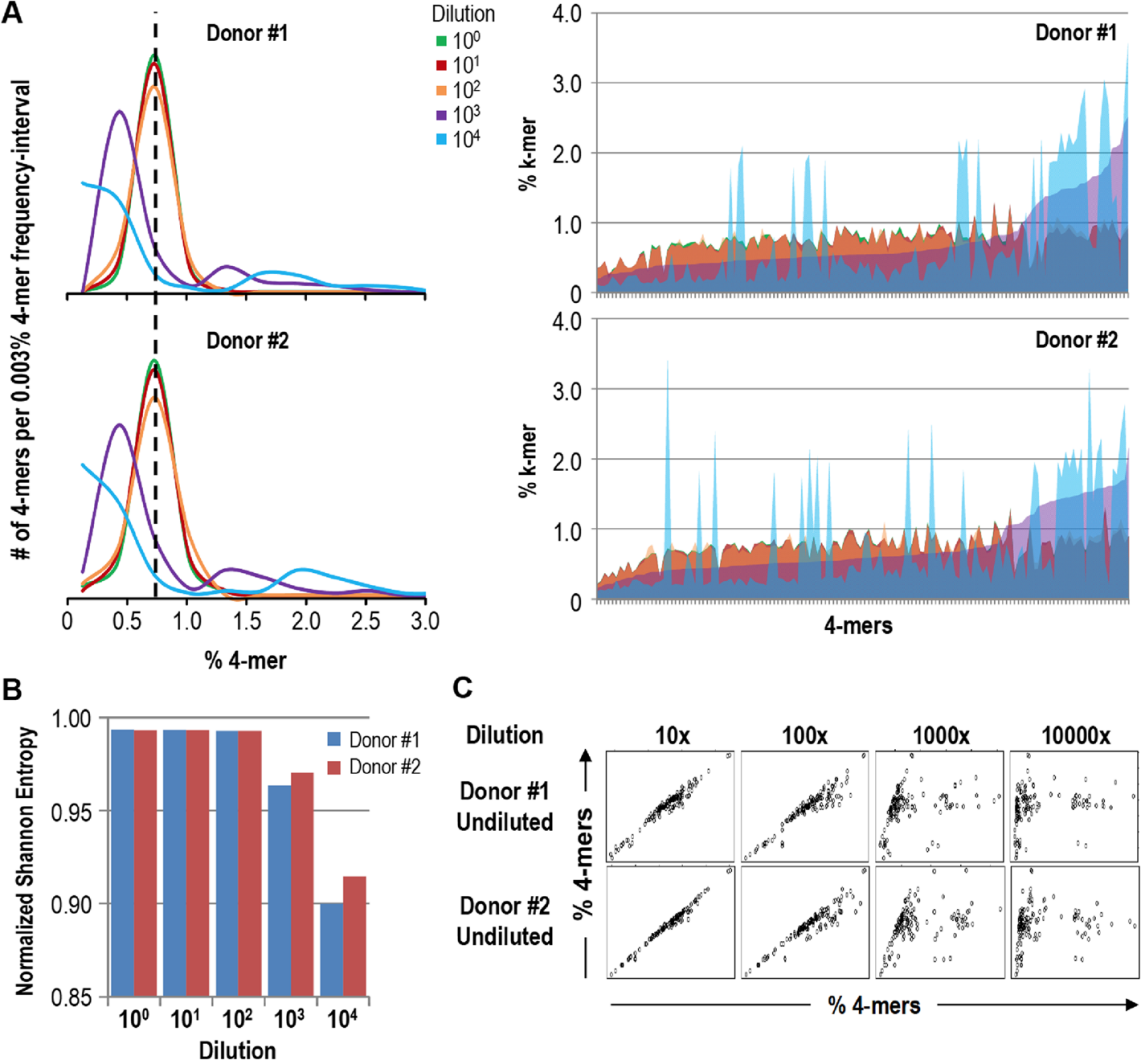
**4-mer distribution analysis of raw metagenomic sequences of serially diluted gut microbiota. A,** 4-mer abundance distribution (left panel) and individual frequency (right panel) of metagenomic sequences from colour coded dilution series metagenomics of gut microbiota from donor #1 (upper panel) and #2 (lower panel). **B,** Bar plot visualizes the normalized Shannon Entropy of 4-mer distribution for undiluted and 10-, 100-, 1000- and 10.000-fold diluted gut microbiota metagenomics from donor #1 (blue) and #2 (red). **C,** Scatter plots depict the correlation between 4-mer distributions of metagenomic sequences from undiluted gut microbiota (y-axis) and 4-mer distributions of metagenomic sequences from 10-, 100-, 1000- and 10.000-fold diluted gut microbiota (x-axis) for donor #1 (upper panel) and #2 (lower panel).

Having established a metagenomic dataset including metagenomes with a defined decline in quality we investigated if k-mer analysis of raw sequences of the same dataset would be able to predict the lower sample size limit as defined in the previous paragraph based on a comparative gene mapping procedure. 4-mer analysis of raw metagenomes corresponding to dilution series samples (1 to 10.000 fold dilutions) of gut microbiota from donor #1 and #2 identified a biased 4-mer distribution for 1.000- and 10.000-fold dilution samples from both donor #1 and #2 (Figure 3A, left panel). Interestingly, aberrant k-mers were not fully overlapping between sample dilutions (Figure 3A, right panel), suggesting that low quality is derived from both sample preparation and system noise. Calculating the Shannon-Entropy for 4-mer distributions from all metagenomes confirmed that the two most dilute samples suffered from a particularly biased raw sequence read composition (Figure 3B). To identify aberrant 4-mers, we correlated the 4-mer frequency observed for each dilution series metagenome with the 4-mer frequency observed for the undiluted reference sample of donor #1 and #2, respectively (Figure 3C). This analysis revealed a distinct subset of 4-mers largely overrepresented in the diluted samples. A closer look at these 4-mers uncovered a tight association with the unique barcode-cassette sequence flanking the genome fragments of the metagenomic shot-gun repertoire. These sequences are derived from self-ligated shot-gun cassettes. Excessive amounts of these sequences are a consequence of limited genomic DNA and subsequent reduced ligation efficiency. Indeed, when we removed all raw sequence reads matching the barcode-cassette sequence of the respective metagenome repertoire, the 4-mer distributions of diluted samples were less aberrant (Additional file 1: Figure S2A), although the 10.000-fold diluted sample remained quantitatively more biased (reduced Shannon-Entropy) than the other dilutions for both donor #1 and #2 (Additional file 1: Figure S2B). Similarly, the correlation analysis revealed that the 10.000-fold diluted sample included k-mers largely overrepresented in the diluted sample compared to the undiluted reference k-mer distribution (Additional file 1: Figure S2C). Of note, this bias is correlated with the skewed gene distribution observed for the 10.000-fold dilution (Figure 2B).

These observations demonstrate that metagenomic quality, as defined by the capacity to precisely and robustly define gene distributions of microbiota, can be predicted by a k-mer distribution analysis of metagenomic raw sequences. It is however not clear if the skewed k-mer distribution observed for the highest sample dilutions (corresponding to low quality metagenomes) is due to aberrant bacterial gene sequences, as observed by correlative analysis of mapped reads (Figure 2), or due to concomitant non-mappable sequences similar to but distinct from the barcode-cassette sequences discussed above. We therefore filtered raw metagenome sequences to only contain mappable sequences. 4-mer analysis revealed an almost equal distribution of 4-mers for all dilution series metagenomes (Additional file 1: Figure S3A) resulting in very similar Shannon-Entropy for 4-mer distributions of all samples (Additional file 1: Figure S3B). Equally, k-mer frequencies correlated perfectly between dilution series samples from the same donor (Additional file 1: Figure S3C). The predictive features of the k-mer analysis are therefore relying on a secondary but concomitant degradation of sequence quality and distribution.

### K-mer distribution predicts metagenomic sequence mapping to a reference gene catalogue

**Figure 4:**
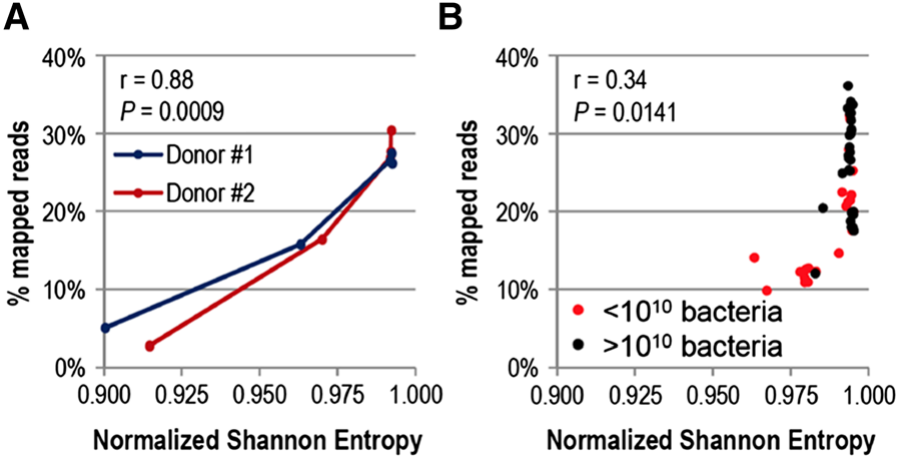
**4-mer distribution of microbiota metagenomes correlates with gene mapping efficiency to a reference gene catalogue. A**, Line graphs depict the frequency of gene mapping to a reference gene catalogue as a function of the normalized Shannon Entropy of 4-mer distributions for undiluted and 10-, 100-, 1000- and 10.000-fold diluted gut microbiota metagenomics from donor #1 (blue) and #2 (red). **B**, Scatter plot illustrates the association between normalized Shannon Entropy of 4-mer distributions and the frequency of gene mapping to a reference gene catalogue for 52 gut microbiota metagenomic profiles stratified according to small (red dots, <10^10^ bacteria) and large (black dots, >10^10^ bacteria) sample size. Spearman rank correlation statistics are indicated.

Our data demonstrate that k-mer analysis is primarily identifying the presence of aberrant sequences, such as contaminations linked to poor metagenome library assembly resulting from limited quantity of genomic DNA. Because sequence contaminations are unlikely to map to known bacterial genes, we speculated that skewed k-mer distributions could predict the frequency of raw sequence reads mapping to the reference gene catalogue. Of note, raw sequences in this context refer to entirely unmanipulated NGS datasets. This approach was chosen to render the methodology broadly applicable. Indeed, we were able to show a clear positive association between 4-mer distribution quantified as Shannon-Entropy and the frequency of mapped reads for dilution series metagenomes of donor #1 and #2 (r = 0.88, *P* = 0.0009 - Figure 4A). Of note, the three most concentrated dilution series samples for both donor #1 and #2 had very similar 4-mer distributions and thus similar gene mapping frequency, whereas the more diluted samples suffered a pronounced drop in the uniformity of their 4-mer distribution with an associated drop in gene mapping efficiency. Applying this analytical approach to a set of 52 metagenomes of 28 human gut microbiota (some gut microbiota were analyzed up to three times with different initial sample size input) showed that our observation was generally applicable, and that 4-mer analysis predicted gene mapping efficiencies below approximately 20% (r = 0.34, *P* = 0.0141 - Figure 4B). Of note, the rate of mapping was based on unfiltered raw sequences and therefore lower than previously reported [32]. We observed that low mapping efficiency was strongly associated with limiting sample material (less than 10^10^ bacteria per sample – Figure 4B). Low (<10^10^ bacteria) and high (>10^10^ bacteria) quantity samples differed significantly with regards to the quantity of DNA available for the ligation step of metagenomic library construction (P = 0.0004; median values and 25%-75% ranges are 1.0 μg [1.0;1.0] and 0.7 μg [0.6;1.0], respectively). The quantities were conform with what was observed for the dilution series samples (cf. Table 1). Above a mapping efficiency of 20% the normalized Shannon Entropy reaches a plateau despite variation in mapping efficiency. This is likely to be a consequence of the relatively large inherent variation in gene distributions between individuals, which is more or less compatible with the known but still incomplete gene reference catalog [4]. The constant increase in gene coverage provided by reference catalogues should eventually remove variations of gene mapping between samples.

## Conclusion

The metagenomic protocol employed in the present study enabled analysis of samples containing more than 10^8^ bacteria (1000-fold dilution). This lower limit fits most live habitat derived microbiota, whereas e.g. analysis of dental plaques from skeletons [8] or other low density microbiota habitats, may be inherently biased in gene and/or species distribution due to limiting sample size. Our study suggests that for these studies it is important to validate the employed metagenomic protocol (e.g. by analyzing a serial dilution of a known quantity of commensals) as described here. Of note, the present study monitors the gene distribution of microbiota. It is likely that reducing the zoom from gene to a given phylogenetic level would equilibrate a large amount of the variance observed at the gene distribution level of low quality metagenomic datasets.

Our study demonstrates that a k-mer distribution analysis of metagenomic raw sequence reads identifies metagenomes of low quality and predicts low gene mapping efficiency. Low quality metagenomes were defined as metagenomes for which the gene distribution was considerably different from a reference sample. In the present study this was modelled by concentrated versus dilute samples of two stool samples. Metagenome quality was lowered by a significant reduction of sample size. It remains to be validated if the technology would also apply to metagenomes suffering from e.g. technical biases or contaminations.

We propose that k-mer analysis of raw metagenome sequence reads should be implemented as a first quality assessment of raw NGS data prior to filtering and gene mapping analysis. It would allow a qualified decision as to whether 1) obtained metagenomic dataset should be further analyzed (filtering, gene mapping etc.), 2) if more sequence reads should be acquired to surpass a predetermined threshold of mapped reads or 3) sample should be discarded or reprocessed to improve metagenomic quality. With the rising demand for metagenomic analysis of microbiota it is crucial to provide tools for rapid and efficient decision making. This will eventually lead to a faster turn-around time, higher quality analysis including measurable quality metrics and a significant cost reduction. Finally, increased quality would have a major impact on the robustness of biological and clinical conclusions drawn from metagenomic studies.

## Additional file

Additional file 1: Table S1. All bacterial genomes can be obtained from NCBI (http://www.ncbi.nlm.nih.gov/taxonomy). **Figure S1.** 4-mer distribution analysis of 26 bacterial genomes and 52 metagenomic sequences of gut microbiota from low and high bacterial content samples. **Figure S2.** 4-mer distribution analysis of barcode-cassette filtered metagenomic sequences of serially diluted gut microbiota. **Figure S3.** 4-mer distribution analysis of gene mapped metagenomic sequences of serially diluted gut microbiota.
