# Supplementary material for: Quality control of microbiota metagenomics by k-mer analysis
Source: BMC Genomics. 2015 Mar 14;16(1):183. doi: 10.1186/s12864-015-1406-7 (PMC4373121; doi:10.1186/s12864-015-1406-7)
Supplement: Additional file 1: Table S1. — All bacterial genomes can be obtained from NCBI (http://www.ncbi.nlm.nih.gov/taxonomy). Figure S1. 4-mer distribution analysis of 26 bacterial genomes and 52 metagenomic sequences of gut microbiota from low and high bacterial content samples. Figure S2. 4-mer distribution analysis of barcode-cassette filtered metagenomic sequences of serially diluted gut microbiota. Figure S3. 4-mer distribution analysis of gene mapped metagenomic sequences of serially diluted gut microbiota. [file 12864_2015_1406_MOESM1_ESM.pdf]

Supplemental table 1

All bacterial genomes can be obtained from NCBI (http://www.ncbi.nlm.nih.gov/taxonomy).

Bacterial strain	Taxonomy_ID
Akkermansia muciniphila ATCC BAA-835	349741
Enterococcus faecalis D32	1206105
Enterococcus faecium DO	333849
Eubacterium rectale ATCC 33656	515619
Lactobacillus brevis ATCC 367	387344
Lactobacillus casei ATCC 334	321967
Methanobrevibacter smithii ATCC 35061	420247
Parabacteroides distasonis ATCC 8503	435591
Roseburia hominis A2-183	585394
Atopobium rimae ATCC 49626	553184
Bacteroides intestinalis DSM 17393	471870
Bifidobacterium angulatum DSM 20098 = JCM 7096	518635
Bifidobacterium bifidum NCIMB 41171	398513
Bifidobacterium longum subsp. longum ATCC 55813	548480
Collinsella aerofaciens ATCC 25986	411903
Collinsella intestinalis DSM 13280	521003
Dorea formicigenerans ATCC 27755	411461
Enterobacter cancerogenus ATCC 35316	500639
Escherichia coli 83972	525281
Faecalibacterium prausnitzii A2-165	411483
Fusobacterium nucleatum subsp. nucleatum ATCC 23726	525283
Fusobacterium periodonticum ATCC 33693	546275
Prevotella copri DSM 18205	537011
Ruminococcus obeum ATCC 29174	411459
Streptococcus salivarius SK126	596322
Salmonella enterica subsp. enterica serovar Choleraesuis str. SC-B67	321314
Bacteroides vulgatus CL09T03C04	997891
Coprococcus comes SL7/1	717958

Supplemental figure legends

Figure S1. 4-mer distribution analysis of 26 bacterial genomes and 52 metagenomic sequences of gut microbiota from low and high bacterial content samples. A, 4-mer abundance distribution of 28 complete bacterial genomes (top panel) and metagenomic sequences of gut microbiota from 21 low ($<10^{10}$ bacteria) (middle panel) and 31 high ($>10^{10}$ bacteria) (bottom panel) bacterial content samples. **B,** Comparison of the median normalized Shannon Entropy of k-mer distributions from the three groups described above with whiskers depicting the 95% confidence interval. P-values are calculated using a non-parametric Mann-Whitney test.

Figure S2. 4-mer distribution analysis of barcode-cassette filtered metagenomic sequences of serially diluted gut microbiota. A, 4-mer abundance distribution (left panel) and individual frequency (right panel) of metagenomic sequences from colour coded dilution series metagenomics of gut microbiota from donor #1 (upper panel) and #2 (lower panel). **B,** Bar plot visualizes the normalized Shannon Entropy of 4-mer distribution for undiluted and 10-, 100-, 1000- and 10.000-fold diluted gut microbiota metagenomics from donor #1 (blue) and #2 (red). **C,** Scatter plots depict the correlation between 4-mer distributions of metagenomic sequences from undiluted gut microbiota (y-axis) and 4-mer distributions of metagenomic sequences from 10-, 100-, 1000- and 10.000-fold diluted gut microbiota (x-axis) for donor #1 (upper panel) and #2 (lower panel)

Figure S3. 4-mer distribution analysis of gene mapped metagenomic sequences of serially diluted gut microbiota. A, 4-mer abundance distribution (left panel) and individual frequency (right panel) of metagenomic sequences from colour coded dilution series metagenomics of gut microbiota from donor #1 (upper panel) and #2 (lower panel). **B,** Bar plot visualizes the normalized Shannon Entropy of 4-mer distribution for undiluted and 10-, 100-, 1000- and 10.000-fold diluted gut microbiota metagenomics from donor #1 (blue) and #2 (red). **C,** Scatter plots depict the correlation between 4-mer distributions of metagenomic sequences from undiluted gut microbiota (y-axis) and 4-mer distributions of metagenomic sequences from 10-, 100-, 1000- and 10.000-fold diluted gut microbiota (x-axis) for donor #1 (upper panel) and #2 (lower panel)

Figure S1

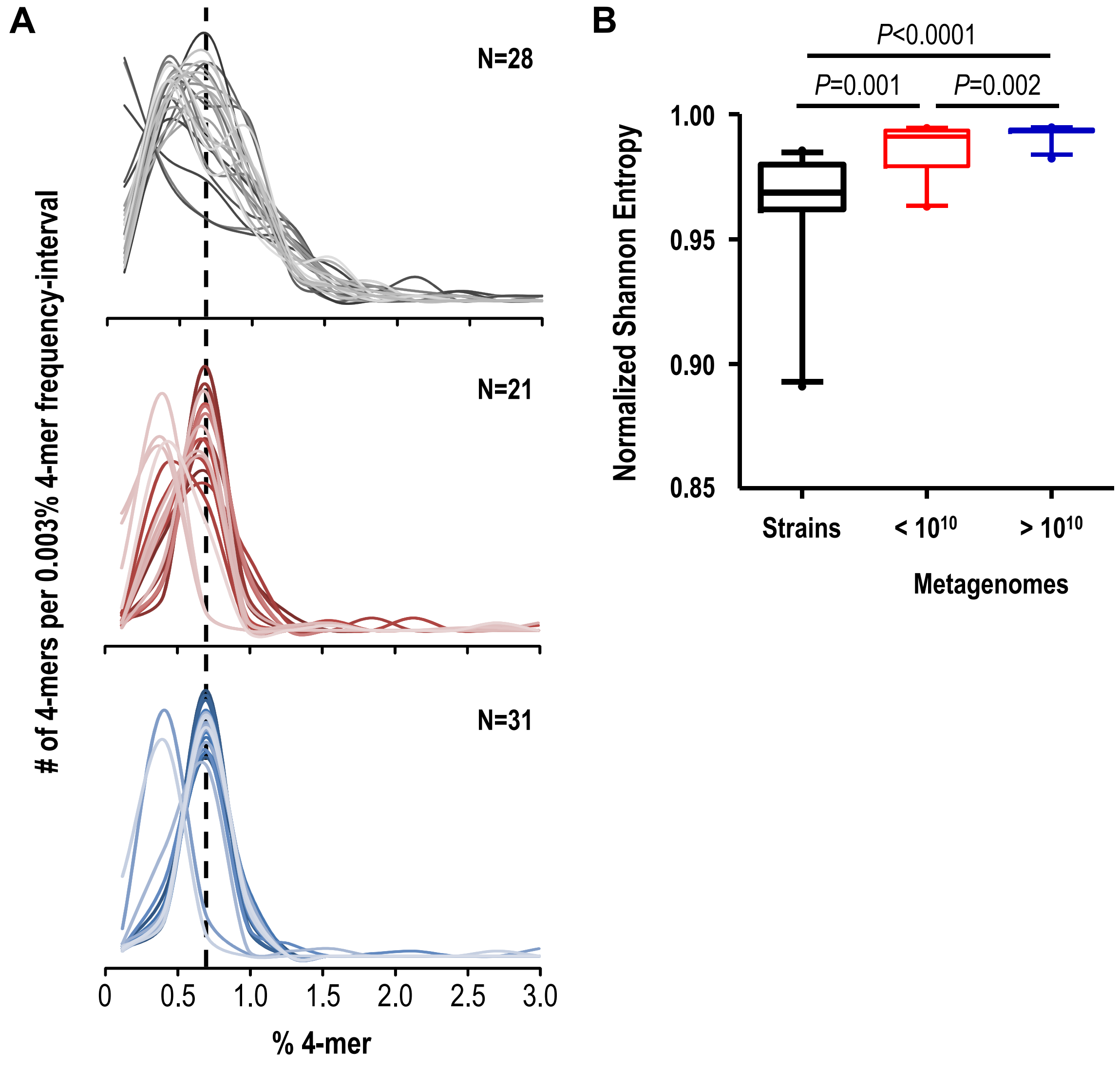
A
N=28
N=21
N=31
# of 4-mers per 0.003% 4-mer frequency-interval
0
0.5
1.0
1.5
2.0
2.5
3.0
% 4-mer
B
P<0.0001
P=0.001
P=0.002
1.00
0.95
0.90
0.85
Normalized Shannon Entropy
Strains
< 10^10
> 10^10
Metagenomes

Figure S2

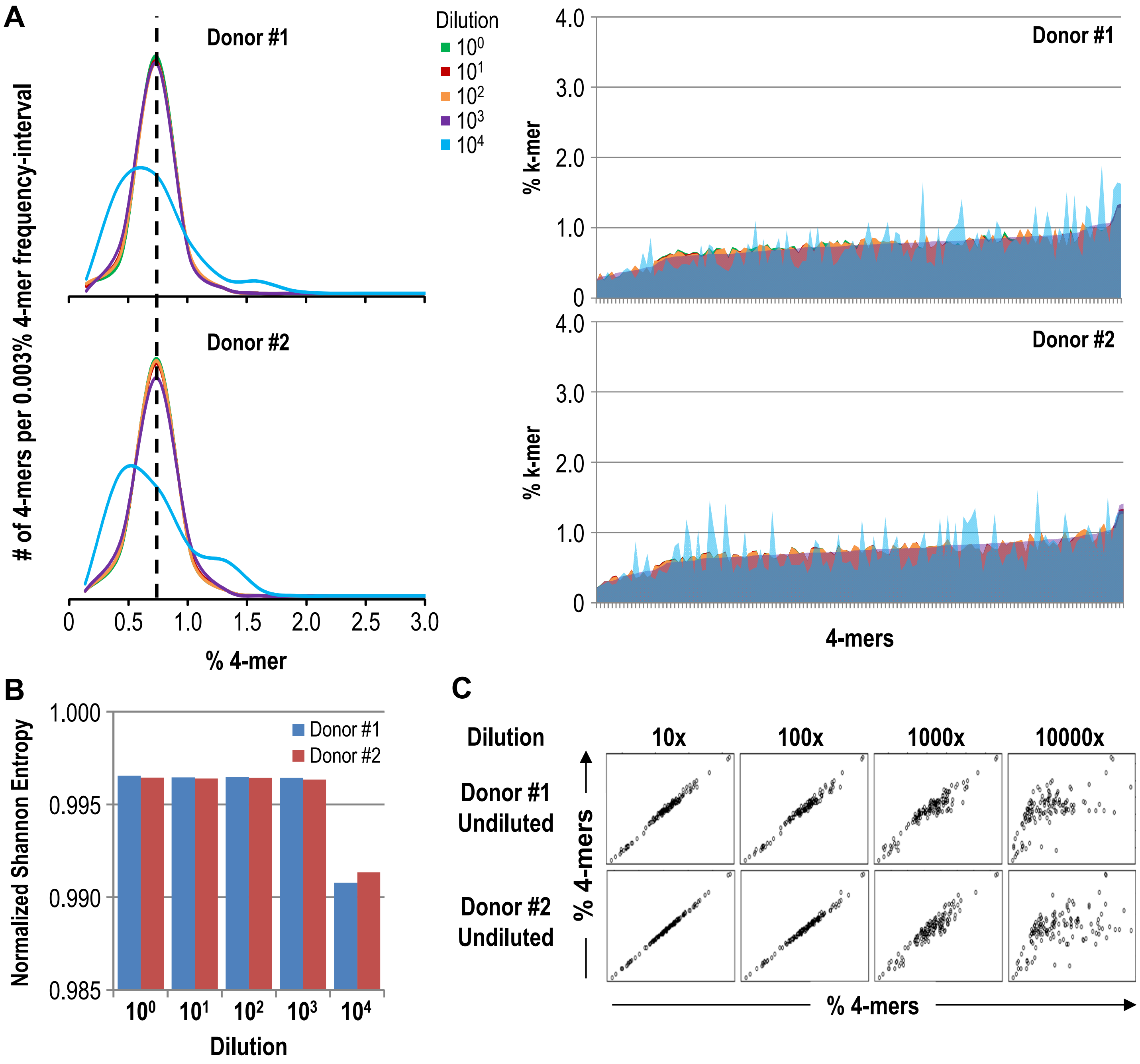
A
Donor #1
Donor #2
Dilution
10^0
10^1
10^2
10^3
10^4
# of 4-mers per 0.003% 4-mer frequency-interval
0
0.5
1.0
1.5
2.0
2.5
3.0
% 4-mer
4.0
3.0
2.0
1.0
0
% k-mer
4-mers
B
1.000
0.995
0.990
0.985
Normalized Shannon Entropy
Donor #1
Donor #2
10^0
10^1
10^2
10^3
10^4
Dilution
C
Dilution
10x
100x
1000x
10000x
Donor #1 Undiluted
Donor #2 Undiluted
% 4-mers
% 4-mers

Figure S3

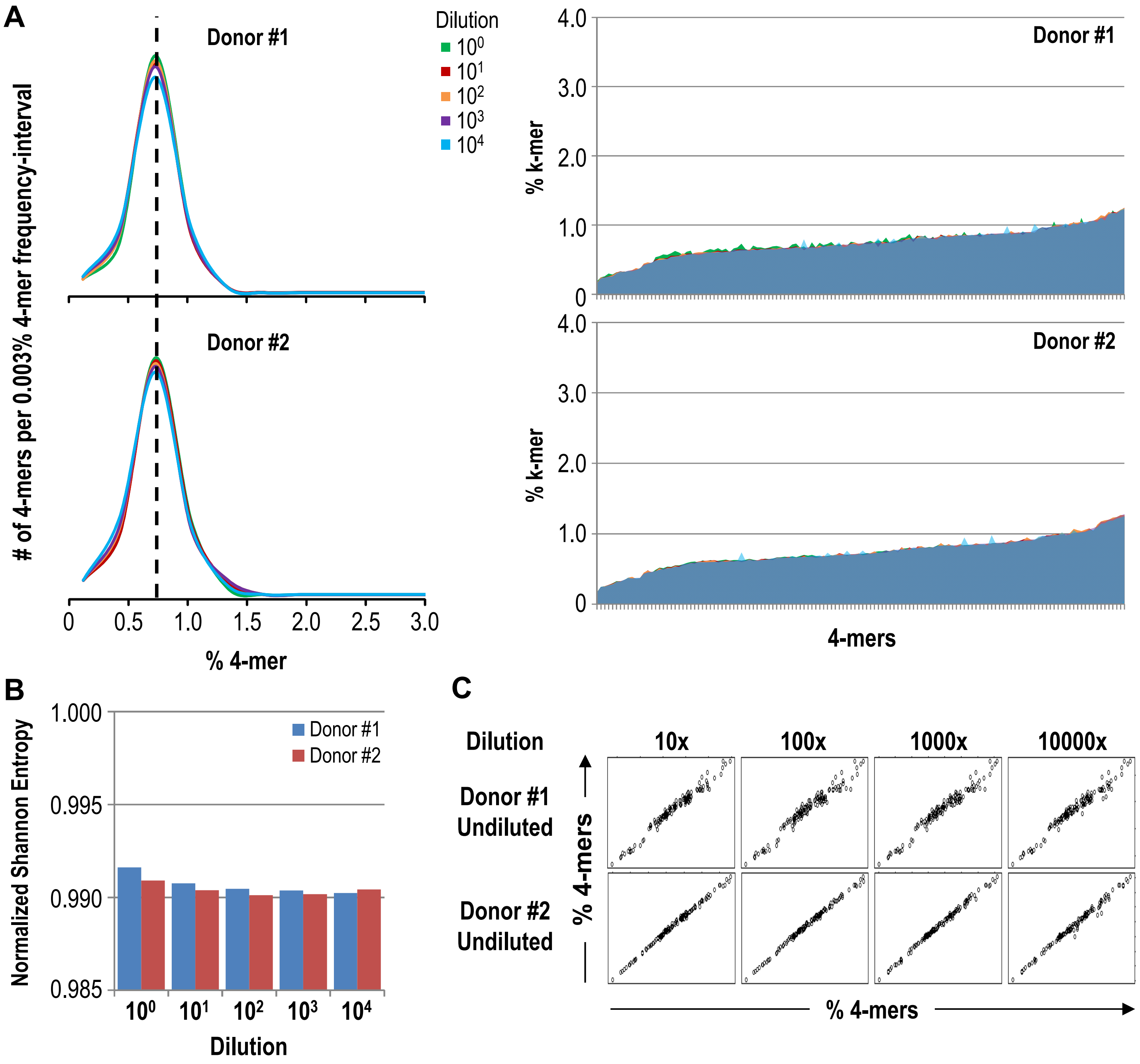
A
Donor #1
Donor #2
# of 4-mers per 0.003% 4-mer frequency-interval
% 4-mer
0
0.5
1.0
1.5
2.0
2.5
3.0
Dilution
10^0
10^1
10^2
10^3
10^4
% k-mer
4.0
3.0
2.0
1.0
0
4-mers
B
Normalized Shannon Entropy
1.000
0.995
0.990
0.985
Donor #1
Donor #2
Dilution
C
Dilution
10x
100x
1000x
10000x
Donor #1 Undiluted
Donor #2 Undiluted
% 4-mers
% 4-mers
